# Production of Domain 9 from the cation-independent mannose-6-phosphate receptor fused with an Fc domain

**DOI:** 10.1007/s10719-024-10169-4

**Published:** 2024-10-09

**Authors:** Yu-He Tang, Yi-Shi Liu, Morihisa Fujita

**Affiliations:** 1 Key Laboratory of Carbohydrate Chemistry and Biotechnology, Ministry of Education, School of Biotechnology, Jiangnan University, Wuxi, Jiangsu 214122 China; 2 Institute for Glyco-core Research (iGCORE), Gifu University, Gifu, 501-1193 Japan

**Keywords:** Mannose-6-phosphate, Cation-independent mannose-6-phosphate receptor, Lysosomal enzyme, N-glycan, Fusion protein

## Abstract

Lysosomal storage diseases (LSDs) are genetic disorders caused by mutations in lysosomal enzymes, lysosomal membrane proteins or genes related to intracellular transport that result in impaired lysosomal function. Currently, the primary treatment for several LSDs is enzyme replacement therapy (ERT), which involves intravenous administration of the deficient lysosomal enzymes to ameliorate symptoms. The efficacy of ERT largely depends on the mannose-6-phosphate (M6P) modification of the N-glycans associated with the enzyme, as M6P is a marker for the recognition and trafficking of lysosomal enzymes. In cells, N-glycan processing and M6P modification occur in the endoplasmic reticulum and Golgi apparatus. This is a complex process involving multiple enzymes. In the trans-Golgi network (TGN), M6P-modified enzymes are recognized by the cation-independent mannose-6-phosphate receptor (CIMPR) and transported to the lysosome to exert their activities. In this study, we used the 9th domain of CIMPR, which exhibits a high affinity for M6P binding, and fused it with the Fc domain of human immunoglobulin G_1_ (IgG_1_). The resulting fusion protein specifically binds to M6P-modified proteins. This provides a tool for the rapid detection and concentration of M6P-containing recombinant enzymes to assess the effectiveness of ERT. The advantages of this approach include its high specificity and sensitivity and may lead to the development of new treatments for LSDs.

**Supplementary Information:**

The online version contains supplementary material available at 10.1007/s10719-024-10169-4.

## Introduction

Lysosomal storage diseases (LSDs) [1] are rare genetic diseases caused by congenital mutations in the genes encoding lysosomal enzymes or proteins involved in the intracellular transport of lysosomal enzymes, resulting in abnormal lysosome function [2, 3]. Currently, various therapies have been developed for LSD, including hematopoietic stem cell transplantation, small-molecule drug therapy, chemical chaperone therapy, gene therapy, and enzyme replacement therapy (ERT) [4]. ERT directly delivers recombinant lysosomal enzymes lacking in patients through intravenous injection to alleviate the symptoms and prevent disease progression. Although not curative, this treatment can ameliorate symptoms in the short term and is widely used for LSD treatment. The lysosomal enzymes used for treatment contain a mannose-6-phosphate (M6P) modification on the N-glycan. The M6P receptor, known as the cation-independent mannose-6-phosphate receptor (CIMPR), captures and delivers the therapeutic recombinant lysosomal enzymes to abnormal lysosomes to resolve the disease. Therefore, the M6P content on the recombinant protein used for treatment is required for drug absorption rate and therapeutic effectiveness [5, 6].

M6P is a modification commonly found on the N-glycan structures of lysosomal enzymes. Newly synthesized proteins undergo N-glycosylation in the endoplasmic reticulum (ER) [7, 8]. In the Golgi apparatus, M6P modification occurs on mannose residues of the oligomannose- and hybrid-type N-glycan structures. This modification involves two important steps. First, the GlcNAc-1-phosphotransferase (GNPT) complex, consisting of GNPTAB (α and β subunits) and GNPTG (γ subunit), selectively recognizes lysine residues arranged in the correct orientation as substrates on the surface of certain proteins [9]. GNPT then transfers one or two GlcNAc-1-phosphate molecules to the mannose residues to form Man-6-P-GlcNAc. Recently, LYSET/GCAF/TMEM251 was identified as an important regulator of GNPT activity [10–12]. Disruption of LYSET can result in mis-localization of GNPT to the lysosome, a reduction of M6P modification, and a decrease in lysosomal protein levels [10–12]. Subsequently, the Man-6-P-GlcNAc structure formed by GNPT is converted into the M6P structure by hydrolysis of the GlcNAc residue, a process mediated by Uncovering enzyme (UCE) [13]. The M6P modification of N-glycoproteins is completed in the TGN and awaits further recognition.

**Fig. 1:**
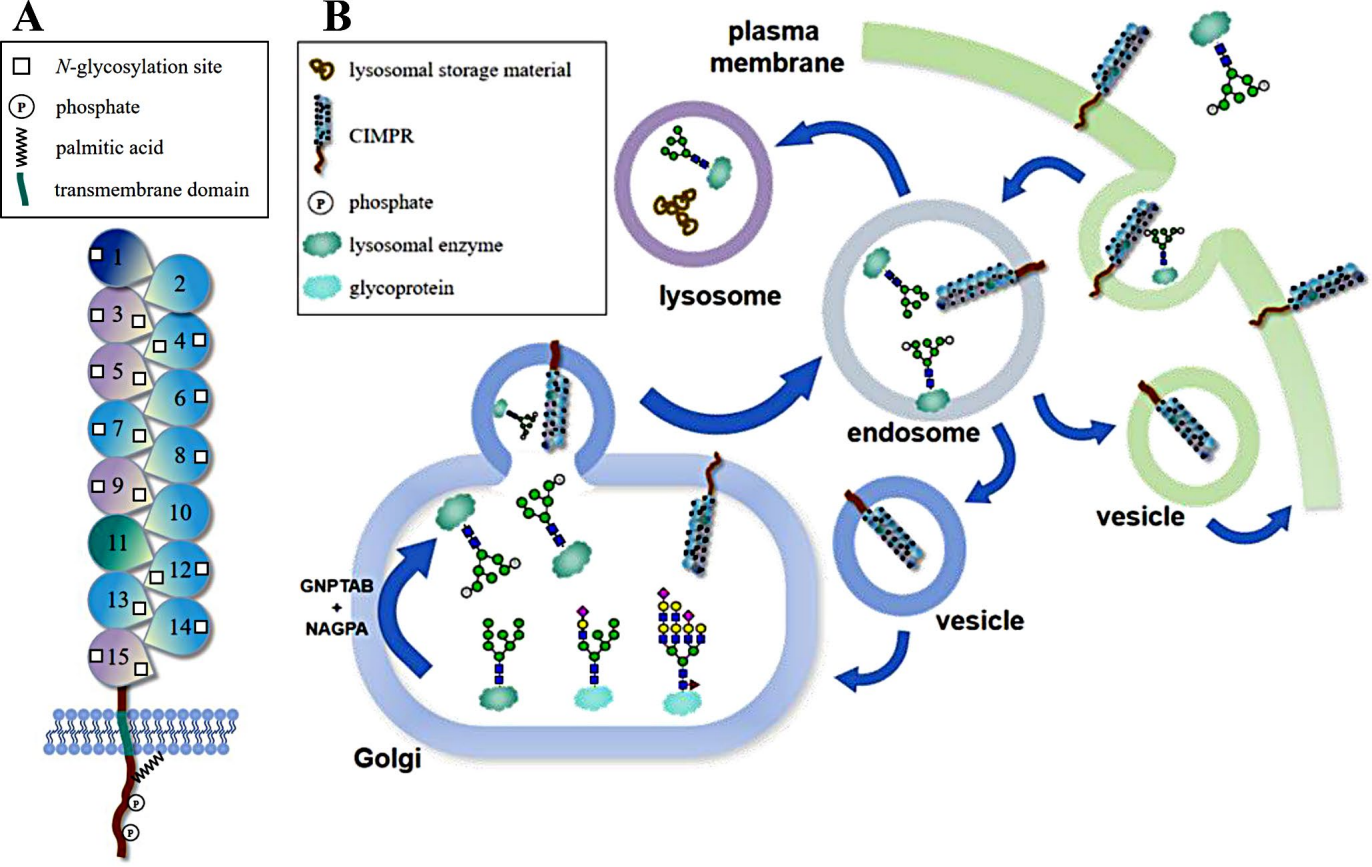
Schematic of mannose-6-phosphate receptor CIMPR and its recognition and transportation function. (**A**) Schematic representation of CIMPR. Domain 1 can bind to urokinase plasminogen activator receptor (uPAR) and plasminogen activator, labeled dark blue; Domain 3, 5, and 9 exhibits M6P recognition activity, marked with purple; Domain 11 has type 2 insulin recognition activity and is marked in green. (**B**) CIMPR is responsible for transporting proteins modified by M6P from the Golgi apparatus or extracellular space to endosomes. Lysosomal proteins are then transported to lysosomes. GlcNAc-1-phosphotransferase (GNPTAB) and N-acetylglucosamine-1-phosphodiester alpha-N-acetylglucosaminidase (NAGPA, also known as the uncovering enzyme, UCE) are enzymes required for M6P formation. This step takes place within the Golgi apparatus

At the TGN, two types of receptors, the cation-dependent M6P receptor (CDMPR) and CIMPR, recognize M6P-containing N-glycoproteins for incorporation into vesicles toward the endosomes. CIMPR, also known as the insulin-like growth factor-II receptor (IGF2R) belongs to the P-type lectin family [14] (Fig. 1A). It is a macromolecular protein (300 kDa) consisting of 15 repetitive domains with ligand-binding sites [15, 16]. Along with its two primary ligands, M6P and IGF-II, CIMPR plays an important role in various cellular processes, particularly protein degradation and transport [17]. The majority of CIMPR is localized in the TGN and the endosome, where it forms dimers on the membrane and interacts with its ligands [18]. Its primary function is to shuttle N-glycoproteins containing M6P moieties to the lysosome. CIMPR recognizes and binds to these specific proteins and transports them from the TGN to the lysosome via endosomes. During this process, the luminal environment gradually becomes acidic [19], leading to the dissociation of CIMPR from M6P. Lysosomal enzymes modified with M6P are further transported to the lysosome, whereas CIMPR returns to the TGN through recycling vesicles or tubules [20] (Fig. 1B). Among the 15 repetitive CIMPR domains, domains 3, 5, 9, and 15 all contain M6P recognition sites [21]. Domain 3 requires the co-expression of Domains 1 and 2 to maintain recognition activity, whereas recognition by Domain 5 is more inclined toward M6P-GlcNAc. Domain 15 binds to both M6P and M6P-GlcNAc with similar affinity [21]. Domain 9, however, has a high affinity for M6P [22], although binding varies with the pH of the environment [23].

In this study, we fused Domain 9 with the Fc domain of human immunoglobulin G_1_ (IgG_1_) antibody to construct a lectin-like Domain 9 for the rapid detection of the M6P modification of recombinant enzymes.

## Materials and methods

### Cells, antibodies, and reagents

HEK293 and HEK293T cells were cultured in Dulbecco’s modified Eagle medium (Biological Industries, Kibbutz Beit Haemek, Israel) supplemented with 10% fetal bovine serum (FBS; Biological Industries). OPTI-MEM and Lipofectamine 3000 were obtained from Thermo Fischer Scientific (Waltham, MA, USA). The His-Trap Ni-NTA column (1 mL, used for the purification of the model protein LIPA) was purchased from Cytiva (Marlborough, MA, USA). Protein A Sefinose Resin 4FF (Settled Resin), bovine serum albumin (fraction V, heat shock isolation), and non-fat powdered milk were obtained from BBI Life Sciences Corporation (Shanghai, China). Horseradish Peroxidase (HRP)-conjugated AffiniPure Goat Anti-Human IgG (H + L) was obtained from Jackson ImmunoResearch (West Grove, PA, USA). PNGase F and Endo H_f_ were procured from New England Biolabs (Ip swish, MA, USA). Jack bean α-mannosidase and mouse anti-FLAG antibodies were purchased from Merck (Rahway, NJ, USA). Alkaline Phosphatase (*E. coli* C75, BAP) was obtained from Takara (Shiga, Japan). D-Mannose-6-Phosphate (sodium salt hydrate) was obtained from Cayman (Ann Arbor, MI, USA). Dimethyl sulfoxide (DMSO), sodium hydroxide pellets, and other chemicals were purchased from Sinopharm Chemical Reagent (Shanghai, China).

### Plasmid construction

A DNA fragment encompassing Domain 9 plus a His6-tag segment was synthesized (Synbio Technologies, Suzhou, China). This fragment was subsequently ligated into the two *XhoI* sites of pME-IgLD, resulting in the generation of pME-Domain9-His6-Fc. After construction, the plasmids were sequenced for confirmation.

### Protein expression and purification

To produce lectin-like Domain 9, pME-Domain9-His6-Fc was transiently transfected into HEK293T cells in 15 cm dishes. The medium was changed after 12 h. The cells were harvested and expanded by dividing them into four 15-cm plates after 24 h. Once the cells were attached to the plates, the medium was changed from DMEM containing 10% FBS to DMEM containing 1% FBS. The medium was collected at 3 days and 5 days of culture, and Domain9-His6-Fc secreted into the medium was purified using Protein A resin.

To produce the model protein LIPA, pHEK-HF-LIPA [24] was transiently transfected into HEK293 cells in 15 cm dishes. The culturing method was the same as that for Domain 9 production. LIPA was secreted into the medium and purified using a His-Trap Ni-NTA column (1 mL).

### Enzyme treatment and western blot analysis

The purified model protein LIPA underwent treatment with PNGase F, Endo Hf, α-mannosidase, and BAP, respectively. Briefly, the purified LIPA samples were diluted in denaturing buffer (0.5% SDS, 40 mM DTT) and heated at 95 °C for 10 min. After cooling to room temperature, the protein samples were treated with PNGase F, Endo Hf, α-mannosidase, or BAP in the proper buffer conditions supplemented for 3 h at 37 °C. A non-enzyme-treated protein sample was also incubated at 37 °C as a control. The samples were subject to SDS-PAGE and transferred to a PVDF membrane. Primary detection of the FLAG-tagged model protein, LIPA, was performed using a mouse anti-FLAG-tag antibody (3000-fold dilution), followed by detection with HRP-conjugated goat anti-mouse IgG (3000-fold dilution). To detect M6P, purified Domain9-His6-Fc was used as the primary antibody (3 µg/ml), followed by detection with HRP-conjugated goat anti-human IgG.

### Immunoprecipitation

HEK293 cells were cultured in 15 cm dishes. The cells (2 × 10^7^ cells) were harvested with trypsin/EDTA, washed with cold PBS at 4 °C, and incubated with 550 µL of lysis buffer (25 mM HEPES, pH 7.4, 150 mM NaCl, 1% NP-40, and protease inhibitor cocktail) on ice for 30 min. After centrifuging at 10,000 × *g* for 10 min at 4 °C, the supernatant was collected and the insoluble fractions were removed. Next, 100 µL of Protein A resin was washed three times with PBS. After pre-mixing with 30 µg of Domain9-His6-Fc for 30 min, the protein A resin was added to the cell lysate and incubated for 1–1.5 h at 4 °C. As a control, the same volume of PBS was added instead of Domain 9. After incubation, the resin was washed three times with PBS. The proteins were eluted in 0.2 M glycine-HCl (pH 2.5) five times, with each elution using 100 µL (resin volume) of 0.2 M glycine-HCl, and collected separately.

For the M6P competitive inhibition test, Domain9-His6-Fc and Protein A resin were co-incubated with the lysate sample as described above. After washing with PBS, the resin was first eluted with 100 µL of M6P (10 mM) 3 times, followed by 0.2 M (pH 2.5) glycine-HCl to release the remaining bound protein. The samples were mixed with SDS sample buffer, heated at 95 °C for 5 min, and analyzed by western blot analysis.

### Proteomic analysis

After immunoprecipitation with Domain9-His6-Fc, the protein bands were visualized by silver staining. The SDS-PAGE gel was compared with the silver-stained and western blot bands. Four gel segments at 70 ~ 100 kDa (D9IP-1), 40 ~ 50 kDa (D9IP-2), 30 ~ 40 kDa (D9IP-3), and 25 kDa (D9IP-4),were excised for mass spectrometry. The excised gel samples were incubated with dithiothreitol and alkylated with iodoacetamide. Then, trypsin (mass ratio of 1:50) was used to digest the denatured proteins at 37 °C for 20 h. After digestion, the resulting peptides (enzyme digestion products) were collected by centrifugation. The samples were desalted, freeze-dried at low temperature, redissolved in 0.1% formic acid solution, and stored at − 20 °C until use.

The redissolved samples were subjected to LC-ESI-MS/MS analysis. Mobile phase A was a 0.1% formic acid aqueous solution, whereas mobile phase B was a 0.1% formic acid acetonitrile solution containing 84% acetonitrile. The column was first equilibrated with 95% mobile phase A. Subsequently, the samples were injected into the Trap column automatically and separated under a gradient condition of 0.5 h. The acquisition of the mass-to-charge ratios of the peptides and their fragments was done as follows: After each full scan, 20 fragment spectra (i.e., MS2 scans) were consecutively collected. The raw files generated from the mass spectrometry runs were searched against a database using Proteome Discoverer 1.4 software to identify the proteins.

## Results

### Expression and purification of lectin-like Domain 9

**Fig. 2:**
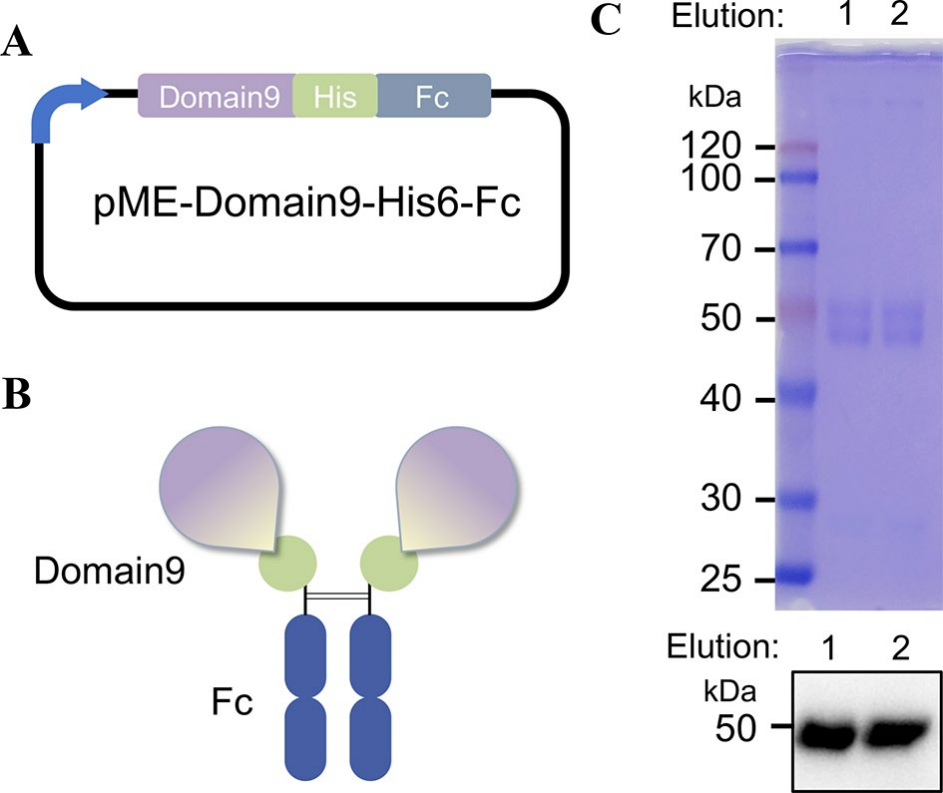
Domain 9 of CIMPR was fused and expressed with the Fc end of human IgG. (**A**) Schematic representation of the Domain 9 of CIMPR fused with a human IgG Fc construct. Components of the construct, including the histidine6 (His6)-tag. (**B**) Schematic representation of the lectin-like Domain9-His6-Fc. (**C**) Expressed and purified Domain9-His6-Fc. Proteins in eluted fractions (Elution 1 and 2) from Protein A beads were detected by CBB staining and western blot using HRP-conjugated anti-human IgG

In this study, we expressed Domain 9 of CIMPR fused with a His6-tag and an Fc fragment of human IgG_1_ at the C-terminus to create a lectin-like domain 9 (hereinafter referred to as Domain9-His6-Fc). This construct facilitates the detection of cells or protein samples (expression plasmid designated pME-Domain9-His6-Fc, Fig. 2A, B). Because there are multiple N-glycosylation sites in CIMPR, two predicted N-glycosylation sites, N1246 and N1312, are located in Domain 9. Based on previous reports on the structure of Domain 9 [9, 25], glycosylation at the N1312 site is important to its activity. To successfully obtain Domain 9 with recognition activity, we selected a human embryonic kidney cell line HEK293T for protein production. HEK293T cells, which are derived from HEK293 cells, stably express E1A protein and SV40 large T antigen. They are characterized by a high transfection efficiency, rapid growth, and ease of culture, making them suitable for the expression and production of mammalian proteins [25]. The plasmid pME-Domain9-His6-IgG constructed above was transiently expressed in HEK293T cells and the culture medium was collected. Purification was conducted using Protein A to target the binding characteristics of the fused Fc fragment, resulting in the isolation of Domain9-His6-Fc (Fig. 2C). The expression of Domain 9 in the cell line enables stable acquisition of human glycan modifications.

### Specific detection of M6P using lectin-like Domain 9

**Fig. 3:**
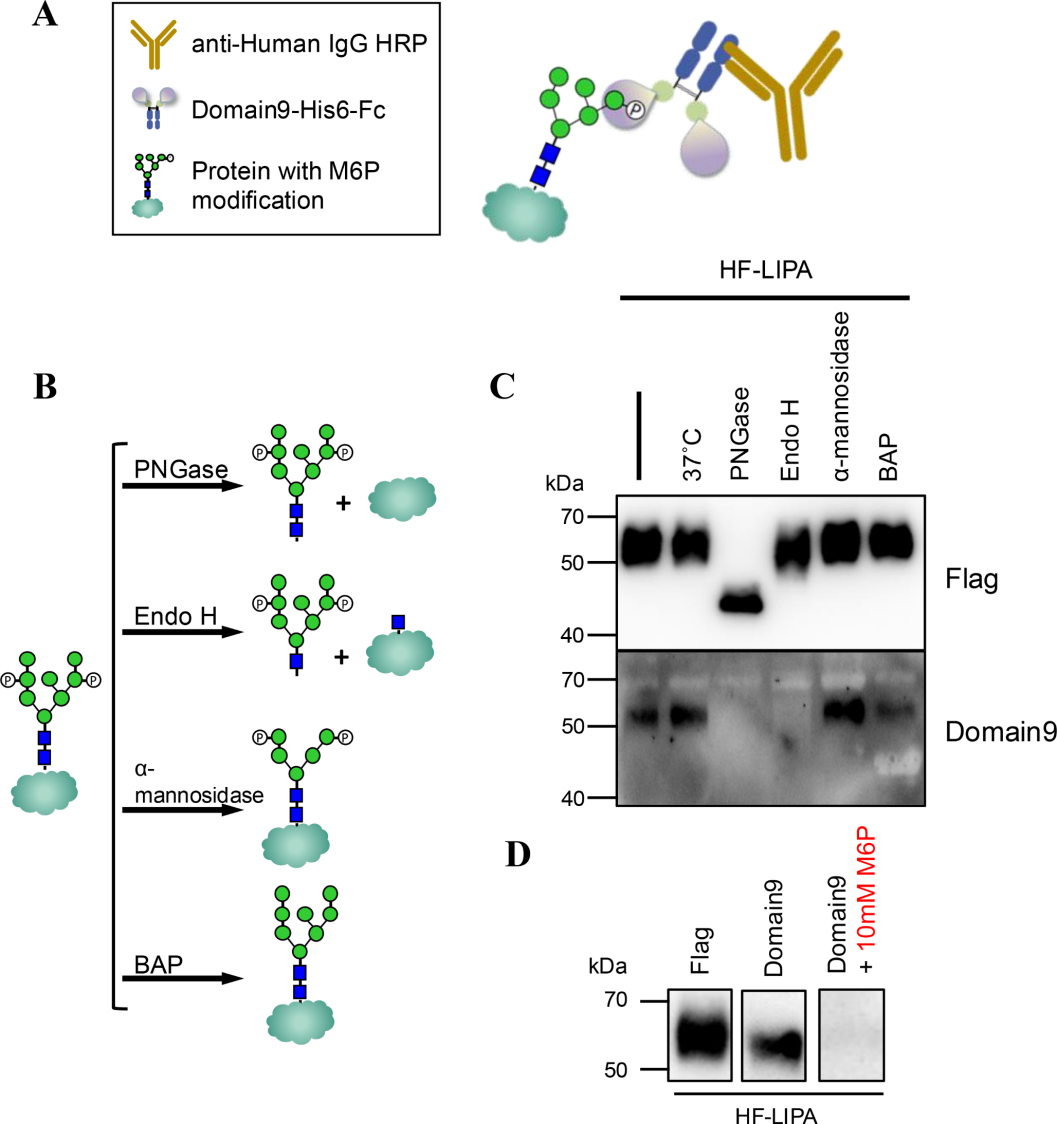
Specific detection of M6P using lectin-like Domain 9 (**A**) Use of lectin-like Domain 9 by western blot analysis. (**B**) The protein His6-Flag-tagged LIPA (HF-LIPA) was treated with glycosidases and phosphatases to verify the recognition activity of Domain 9, respectively. (**C**) Anti-Flag antibody was used to detect the HF-LIPA, whereas Domain 9 was used to detect the glycans on HF-LIPA with various treatment. (**D**) Phosphorylated monosaccharides (10 mM M6P) were added when Domain 9 was used as a primary antibody for western blot analysis to verify the specificity for M6P

To determine whether the purified Domain9-His6-Fc recognizes M6P (Fig. 3A), lysosomal acid lipase (LIPA) was used as a model protein, which contains six N-glycosylation sites [24, 26, 27]. Several N-glycosylation sites on LIPA were modified with M6P (Fig. 3B). By transiently transfecting the plasmid pHEK293-HF-LIPA into wild-type HEK293 cell lines, His6-FLAG-tagged LIPA (HF-LIPA) proteins were collected from the culture medium. The LIPA protein was purified using a Ni^2+^ column and determined to be 50–70 kDa using anti-FLAG antibody (Fig. 3C). When the same sample was detected using Domain9-His6-Fc as the primary antibody, bands at 50 kDa were observed, suggesting that recombinant LIPA contains M6P-containing glycans. The N-glycans were removed from LIPA using two exoglycosidases, PNGase F and Endo H. PNGase F cleaves all types of mammalian N-glycan modifications on proteins, whereas Endo H hydrolyzes the oligomannose-type and hybrid-type N-glycans, but not the complex-type N-glycans (Fig. 3B). The LIPA protein bands migrated faster and were detected around 45 kDa after PNGase F treatment, indicating that the original band dispersion was the result of the heterogeneity in the N-glycan structures. On the other hand, the LIPA band was only mildly shifted when treated with Endo H, suggesting that the fractions of recombinant LIPA contained complex-type N-glycans (Fig. 3C). However, LIPA was not detected using Domain9-His6-Fc following treatment with PNGase F and Endo H (Fig. 3C). This indicates that Domain 9 specifically recognizes M6P in the oligomannose-type and hybrid-type N-glycans, which are sensitive to Endo H. Jack bean α-mannosidase is an exomannosidase that catalyzes the hydrolysis of α-1,2/1,3/1,6-linked mannose at the non-reducing end (Fig. 3B). LIPA detection by Domain9-His6-Fc was relatively increased (Fig. 3C), which suggests that Domain 9 recognition was improved when M6P was exposed at the non-reducing end. BAP is an alkaline phosphatase that catalyzes the hydrolysis of all phosphate monoesters (Fig. 3B). BAP, commonly used for DNA dephosphorylation, was applied here to remove the 6-phosphate modification from mannose. LIPA recognition by Domain9-His6-Fc was decreased following BAP treatment (Fig. 3C) because the phosphate groups were hydrolyzed. However, the band did not disappear completely, suggesting that the dephosphorylation activity may not have been sufficient to remove all phosphorylation sites from M6P.

To determine the specificity of Domain 9 for M6P, Domain 9 was co-incubated with phosphorylated monosaccharide, M6P, during the primary antibody reaction of a western blot analysis. Purified LIPA was detected by both the anti-FLAG antibody and Domain9-His6-Fc; however, the bands were nearly invisible (Fig. 3D), indicating that Domain9-His6-Fc can be used as a primary antibody for the detection of proteins containing M6P-glycans.

### Detection of immunoprecipitation activity using Domain 9

**Fig. 4:**
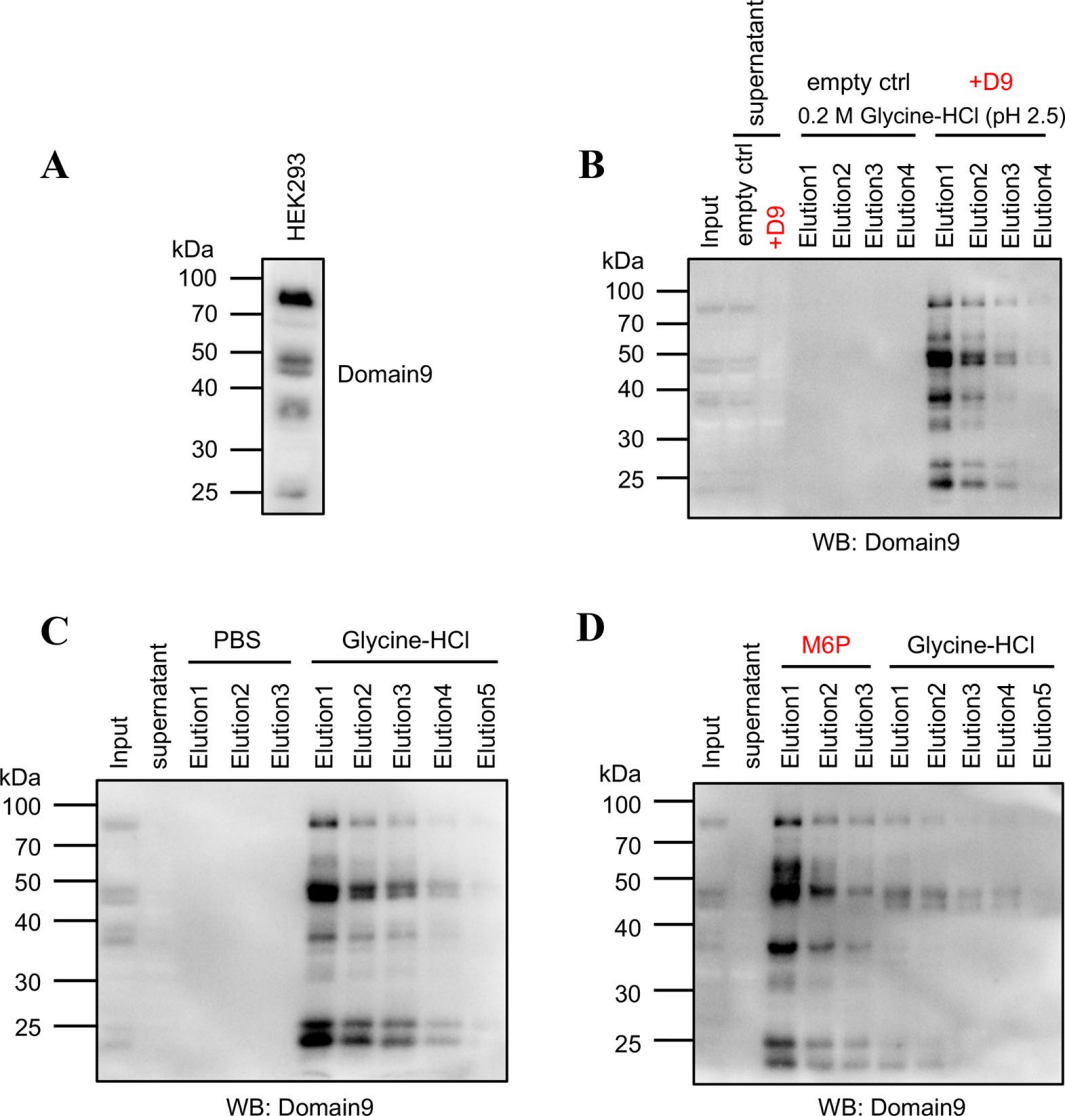
Detection of cell lysate by Domain 9 (**A**) HEK293 cell lysates were subjected to western blot analysis using Domain 9 as the primary antibody, followed by HRP-conjugated anti-human IgG. (**B**) Domain9-His6-Fc (D9) was used to enrich the M6P-containing proteins from HEK293 cell lysate. An equivalent amount of PBS was used as a control. Bound proteins were eluted by 0.2 M glycine-HCl (pH 2.5). Eluted proteins were detected using Domain 9 as the primary antibody. (**C**) Proteins bound to Domain9-His6-Fc and Protein A were eluted using PBS, followed by 0.2 M glycine-HCl (pH 2.5). (**D**) Proteins bound to Domain9-His6-Fc and Protein A were eluted using M6P, followed by 0.2 M glycine-HCl (pH 2.5). Eluted proteins were detected using Domain 9 as the primary antibody

After determining the specificity of Domain 9, we examined whole-cell lysates to assess the levels of M6P in the endogenous proteins within cells. Protein blotting revealed that endogenous M6P-containing glycoproteins were detected at various sizes, ranging from approximately 25 kDa to nearly 100 kDa (Fig. 4A). However, the number of major immunoreactive bands is smaller compared to the number of lysosomal proteins. A previous study reported that lysosomal enzymes with more M6P modifications show enhanced recognition during endocytosis experiments [28], suggesting that proteins with higher levels of M6P modifications are more efficiently recognized by Domain9. To further identify the endogenous proteins that can be recognized, we performed immunoprecipitations from whole-cell lysates using Domain9-His6-Fc and Protein A agarose beads. Cell lysates were incubated with or without Domain9-His6-Fc followed by Protein A agarose. The bands detected by Domain9 disappeared in the supernatant following Protein A agarose precipitation with Domain9-His6-Fc (Fig. 4B). Instead, the Domain 9-enrich *e* d proteins were eluted by the addition of 0.2 M glycine-HCl (pH 2.5). The clearance from the supernatant and elution did not occur in the control without Domain9-His6-Fc, indicating that M6P-containing glycoproteins are bound and pulled down only when Domain 9 is added.

After cell lysates were incubated with Domain9-His6-Fc and Protein A, the M6P-containing glycoproteins were eluted under acidic conditions with 0.2 M glycine-HCl (pH 2.5), but not PBS as a control (Fig. 4C). In contrast, M6P-containing glycoproteins bound with agarose beads were successfully eluted in PBS containing 10 mM free M6P (Fig. 4D). Subsequent elution with glycine-HCl revealed that most of the M6P-containing glycoproteins were eluted with free M6P. The results indicate that protein samples were enriched through immunoprecipitation with Domain9-His6-Fc.

### Identification of endogenous proteins detectable by Domain 9

**Fig. 5:**
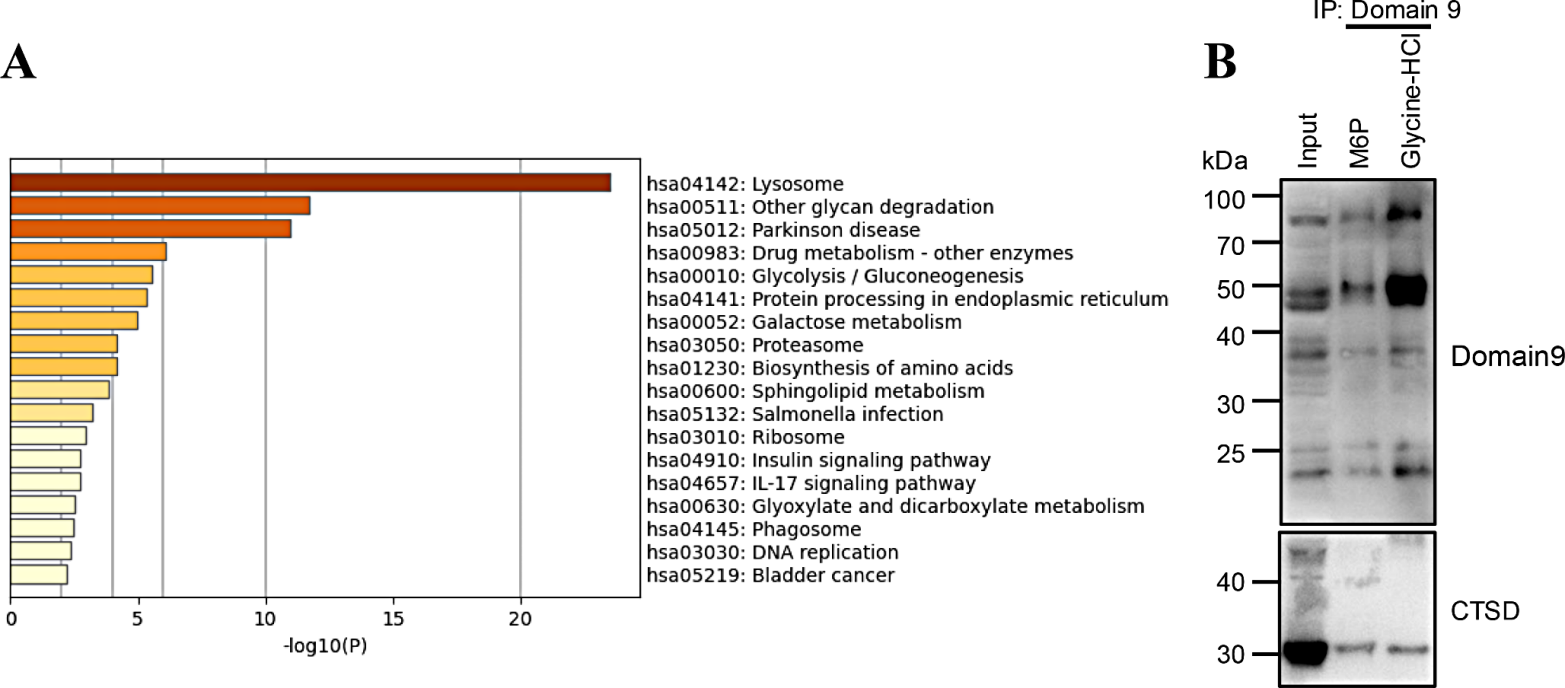
Lysosomal proteins were enriched by Domain 9 (**A**) KEGG analysis of the proteins enriched by Domain 9. Lysosomal proteins detected by MS analysis are shown in Supplementary Table 1. (**B**) Proteins bound to Domain 9 were eluted by M6P or 0.2 M glycine-HCl (pH 2.5). Proteins were detected using either Domain 9 or anti-CTSD antibody

Because of the numerous bands obtained in the samples, we identified specific endogenous proteins that are recognized by Domain 9. Proteins were immunoprecipitated by Domain9-His6-Fc/Protein A agarose and eluted with 0.2 M glycine-HCl (pH 2.5) as above, then detected by both western blot and silver staining (Supplementary Fig. 1). The protein samples were analyzed by liquid chromatography-tandem mass spectrometry (LC-MS/MS). The resulting data were analyzed using mass spectrometry matching software, such as MASCOT, to obtain qualitative identification information for the target proteins. A total of 3,397 different peptides were detected and 879 proteins were analyzed and matched. After the removal of potentially contaminating proteins associated with RNA and keratin, 660 proteins were identified in the sample. Furthermore, Metascape was used to analyze the list of qualified proteins [29], which were subject to KEGG analysis (Fig. 5A). We found that most of the enriched proteins originated from the lysosomes as evidenced by 296 proteins related to lysosomal function and 45 associated with functional lysosomal enzymes (Supplementary Table 1). However, in the lysosomal proteins, non M6P-tagged lysosomal membrane proteins like LAMP1 and LAMP2 were detected in the domain 9-His6-Fc-fragment precipitates. The detected LAMP1 and LAMP2 are likely contaminants in the experiment. There are some known lysosomal enzymes that are not included in the list. The potential reasons for this may be low or no expression in HEK293 cell lines, as well as the loss of samples during preparation and library searching.

To validate the result, we detected a lysosomal protein, CTSD, which was observed in the MS analysis. Proteins immunoprecipitated from cell lysates by Domain9-His6-Fc/Protein A agarose and eluted with either 10 mM free M6P or 0.2 M glycine-HCl (pH 2.5). In both eluates, CTSD was detected (Fig. 5B). Compared to the input fraction, CTSD levels in M6P and glycine-HCl fractions were low probably due to the dephosphorylation of N-glycans on matured CTSD in lysosomes. As shown in the input fraction, the major form of CTSD in the cell lysate is the mature form, while the pro-form is present at very low levels. One possible explanation is that the transport rate of pro-form CTSD with M6P modification is very fast, and it is quickly cleaved into the mature form after being transported to the endosomes/lysosomes. Additionally, some pro-form proteins may be secreted outside the cell. As a result, the mature form of CTSD that still possesses M6P was predominantly detected in the cell lysates by immunoprecipitation with Domain9, while the pro-form was almost undetectable. These results indicate that Domain9-His6-Fc is useful to enrich M6P-containing lysosomal proteins.

## Discussion

For many LSDs, ERT effectively alleviates symptoms and significantly improves patients’ quality of life. Thus, it is currently the preferred therapeutic regimen for these patients. During the treatment process, the absorption rate of the recombinant therapeutic lysosomal protein is an important factor. Most of the recombinant lysosomal enzymes used for ERT enter the cell through the M6P receptor CIMPR located on the cell surface. Therefore, monitoring M6P levels of recombinant lysosomal proteins is important. Since detecting M6P content is essential for the application of recombinant lysosomal enzymes, various proteins and materials have been developed and used to detect and enrich M6P-containing proteins. For example, CIMPR has been used to purify M6P-containing glycoproteins from rat tissue [30], and His6-tagged Domain9 has been employed for detecting M6P-containing glycoproteins [31–33]. Additionally, previous studies utilized phage display technology to screen and enrich specific antibody fragments (scFv), leading to the construction of ScFv M6P-1, which recognizes the M6P structure [34, 35]. Fe^3+^-IMAC has also been used to enrich M6P-containing proteins from HeLa and CHO cells, in combination with mass spectrometry for glycoproteomic and glycomic analysis [36]. In the method, 35 glycoproteins with M6P were enriched in HeLa cells, and 15 glycoproteins in CHO cells.

In the present study, we focused on the ninth domain of CIMPR for the detection of M6P-containing glycoproteins. We fused Domain 9 with an Fc fragment of human IgG_1_ and expressed it in the HEK293T cell line. This provides a material basis for subsequent biological applications, including the detection and enrichment of M6P-containing glycoproteins. This fusion protein preserves the M6P recognition activity of Domain 9 and the lectin-like structure renders it a convenient detection tool to examine the M6P structure, and offers several advantages compared with His6-tagged Domain 9 and other probes like ScFv M6P-1 and Fe^3+^-IMAC. First, the Domain9-His6-Fc can be purified with a single step using Protein A beads. Second, the Fc-fused proteins improve the avidity for the recognition of M6P, because the binding affinity of lectin against sugars is generally low. CIMPR is often expressed in the form of dimers and located on the membrane [37]. The Fc region would provide a natural dimeric form for the expression of Domain 9, enabling it to fully exert its activity. Therefore, Domain9-His6-Fc could be used not only for the detection of M6P-containing glycoproteins, but also for the immunoprecipitation of these proteins. Subsequently, we used this lectin-type Domain 9 for protein blot detection. By treating purified lysosomal enzymes with glycosidases or a phosphatase, the lectin-like Domain 9 exhibited a high degree of specific recognition for M6P-containing glycoproteins, which provides strong support for its application in monitoring M6P content on glycoproteins. Through immunoprecipitation analysis, we successfully isolated proteins that specifically bind to lectin-type Domain 9. A proteomic analysis was performed to identify these proteins using mass spectrometry and we obtained a list of various lysosomal enzymes.

In our experiment aimed at enriching proteins modified with M6P, we successfully enriched over forty lysosomal enzymes. In the Western blot analysis, only the major protein bands were preferentially detected, whereas we identified over 40 lysosomal proteins in the precipitation analysis. These results indicate that Domain9-His6-Fc recognizes not only abundant proteins but also less abundant proteins with M6P modifications. However, it is known that there are more than sixty lysosomal enzymes, but a dozen of them were not detected in this study. Several factors may explain this. First, we used HEK293 cells as samples, and some lysosomal enzymes, such as CLN5 and GalC, are either expressed at low levels or not expressed at all in these cells. Second, lysosomal enzymes are only transiently modified with M6P during their transport from the TGN to the endosome. By the time they reach the endo/lysosomes, the phosphorylation modification has been removed, which may result in the failure to enrich some highly mobile proteins. Lastly, we obtained samples by cutting gels based on comparisons between silver staining and western blot results, which may have caused some loss of weakly recognized or larger mass proteins (e.g., MAN2B1 and MAN2B2). It is important to note that the primary objective of this enrichment experiment was to demonstrate the specificity of Domain9-His6-Fc for M6P and its potential applications, rather than to enrich all lysosomal enzymes present in HEK293 cells. This study primarily emphasizes the production and specificity testing of the detection tool, Domain9-His6-Fc. In future experimental planning, we can utilize samples with higher biological value, such as plasma or cerebral spine fluid, in conjunction with glycoproteomic mass spectrometry detection methods, to explore therapeutic options for lysosomal storage diseases and identify more potential biomarkers.

In summary, we successfully expressed Domain 9 of CIMPR fused with the Fc fragment in HEK293T cells. The Domain9-His6-Fc exhibited excellent specificity for M6P and may be used to detect or purify proteins containing M6P. This tool will be useful for monitoring the quality of therapeutic recombinant lysosomal enzymes. In addition, it will provide further insight into the biology of M6P-modified glycans and glycoproteins. It may be used to analyze and compare the status of M6P levels in lysosomal proteins in various glycoengineered cells (Ref: Dev Cell and Tang et al., unpublished). In the future, the potential applications of Domain 9 will contribute to human health and lysosomal disease treatment.

## Electronic supplementary material

Below is the link to the electronic supplementary material.

Supplementary Material 1

Supplementary Material 2

## Data Availability

No datasets were generated or analysed during the current study.
