# Correction: Production of Domain 9 from the cation-independent mannose-6-phosphate receptor fused with an Fc domain

**DOI:** 10.1007/s10719-024-10170-x

**Published:** 2024-11-04

**Authors:** Yu-He Tang, Yi-Shi Liu, Morihisa Fujita

**Affiliations:** 1 Key Laboratory of Carbohydrate Chemistry and Biotechnology, Ministry of Education, School of Biotechnology, Jiangnan University, Wuxi, Jiangsu 214122 China; 2 Institute for Glyco-core Research (iGCORE), Gifu University, Gifu, 501-1193 Japan

**Correction to: Glycoconjugate Journal**

10.1007/s10719-024-10169-4

In this article, the current affiliation of the corresponding author is listed as only affiliation 2, however affiliation 1 should also be included.

The original article has been corrected.

