# Supplementary material for: Production of Domain 9 from the cation-independent mannose-6-phosphate receptor fused with an Fc domain
Source: Glycoconj J. 2024 Oct 9;41(6):395–405. doi: 10.1007/s10719-024-10169-4 (PMC11735522; doi:10.1007/s10719-024-10169-4)
Supplement: Supplementary file 2 — Supplementary Material 2 [file 10719_2024_10169_MOESM2_ESM.pdf]

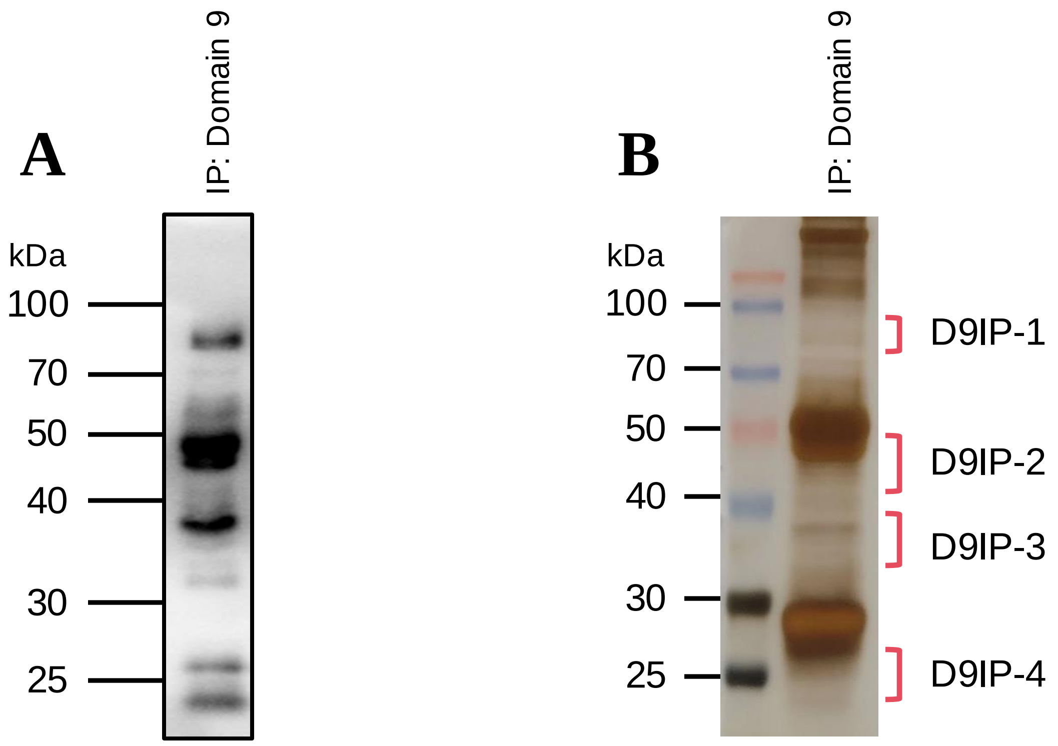

Supplementary Figure 1. Immunoprecipitation from whole cell lysate using Domain9-His6-Fc/Protein A.

A. Western blot analysis of immunoprecipitated sample by Domain9.

B. Silver-stained sample was sectioned as 4 pieces and divided into 3 groups before they were sent for LC-MS analysis.
